# Psychosocial Profile of Bullies, Victims, and Bully-Victims: A Cross-Sectional Study

**DOI:** 10.3389/fped.2014.00001

**Published:** 2014-01-14

**Authors:** Marie Leiner, Alok Kumar Dwivedi, Maria Theresa Villanos, Namrata Singh, Dan Blunk, Jesus Peinado

**Affiliations:** ^1^Department of Pediatrics, Texas Tech University Health Sciences Center, El Paso, TX, USA; ^2^Division of Biostatistics and Epidemiology, Department of Biomedical Sciences, Texas Tech University Health Sciences Center, El Paso, TX, USA; ^3^Department of Medical Education, Paul L. Foster School of Medicine, El Paso, TX, USA

**Keywords:** bullies, victims and bully-victims, children, psychosocial profile

## Abstract

While adverse conditions in a child’s life do not excuse inappropriate behavior, they may cause emotional and behavioral problems that require treatment as a preventive measure to reduce the likelihood of bullying. We aimed to identify differences in the psychosocial profiles of adolescents who classified themselves as bullies, victims, or bully-victims. We performed a cross-sectional study in which data were collected between January 2009 and January 2010 from seven university-based clinics in a large metropolitan area with a predominantly Mexican-American population. We collected data on physical aggression among adolescents who self-categorized into the following groups: uninvolved, bullies, victims, and bully-victims. We determined the psychosocial profiles of the adolescents based on responses to the Youth Self Report (YSR) and parent’s responses to the Child Behavior Checklist (CBCL). A one-way analysis of variance and multivariate regression analyses were performed to compare the various components of the psychosocial profiles among the groups. Our analysis of the CBCL and the YSR assessments identified differences between the uninvolved group and one or more of the other groups. No significant differences were observed among the bully, victim, and bully-victim groups based on the CBCL. We did find significant differences among those groups based on the YSR, however. Our results suggest that emotional and behavioral problems exist among bullies, victims, and bully-victims. Therefore, treatment should not focus only on the victims of bullying; treatment is equally important for the other groups (bullies and bully-victims). Failure to adequately treat the underlying problems experienced by all three groups of individuals could allow the problems of bullying to continue.

## Background

The bullying epidemic persists among children and adolescents despite the substantial investment of interest, programs, and resources intended to eradicate it (1–4). Only a few programs are moderately successful, and their effectiveness is often challenged by new or recurrent acts of bullying (5). A systematic review of 26 years of school-based programs indicated a 20–23% reduction in bullying and a 17–20% reduction in victimization (6). While most preventive efforts concentrate on anti-bullying programs, it might be necessary to consider looking at this phenomenon from a psychosocial and behavioral perspective. Such an approach might reveal the deepest roots of the problem in order to provide more effective solutions.

Children and adolescents can be perpetrators or victims of bullying, and some children fit both roles (bully-victims). Generally, from an emotional and behavioral perspective, victims experience emotional problems, bullies experience behavioral problems, and bully-victims experience both emotional and behavioral problems (7). The bully-victims generally experience the most problems and have the highest risk of adverse outcomes (8–10). Although it is not certain whether bullying is a *cause or an effect* of the emotional and behavioral problems experienced by children, an association between bullying behavior and such problems seems likely; in which case those involved in bullying, whether they are victims or perpetrators, may require comprehensive treatment to solve their emotional and behavioral problems and improve their outcomes in relation to bullying prevention programs. Thus, the lack of treatment for all those involved in acts of bullying may explain why the outcomes of bullying interventions are unclear and hard to measure. Additionally, the success of bullying intervention is limited by the fact that bullying is often recurrent, and any reduction in bullying achieved by an intervention is only temporary.

Detecting emotional and behavioral problems among bullies, victims, and bully-victims is very challenging, because the roles can be interchangeable (11–13). For example, aggressive behavior is rarely a spontaneous behavior that appears without any connection to previous or parallel experiences involving some type of victimization. Some studies indicate that victimization and perpetration are interconnected: bullies are, or have been, victims; and victims are, or will be, potential bullies (14, 15). Evidence suggests that the overlaps between different forms of violence, even those that seem unrelated, are so deep that it can be difficult to identify individuals who have experienced, or perpetrated, only a single form of violence (16, 17). Bullying can be a response to a large and varied assortment of different forms of interpersonal violence and abuse including victimization, neglect, maltreatment, and others (18, 19). As a result of this interchange of roles, identifying problems can be confounded, depending on the role that the child or adolescent has at the moment of responding to an assessment. Moreover, the parents/guardians might look at their behavior differently than the child or adolescent when responding to the same assessment.

This multi-factorial perspective on the measurement of emotional and behavioral problems makes it necessary to use broader standardized instruments to collect information from children and parents about the psychosocial profiles of bullies, victims, bully-victims, and uninvolved individuals (20, 21). Most prior studies considering the psychosocial and behavioral problems of adolescents identified as bullies (9, 22), victims (23), or bully-victims (8) collected data using student surveys (24), national surveys (25, 26), or shorter versions of screening assessments (27). We aimed to extend the scarce literature on the emotional and behavioral profiles of adolescents who classified themselves as bullies, victims, or bully-victims by comparing the responses of the adolescents with those of the parents. In addition, scarce research has been conducted that focuses on children living in poverty, Latinos, and other minorities, which is essential to plan for these populations’ present and future needs. Therefore, this study was conducted among children and adolescents of Mexican-American origin living in poverty, the largest sub-group of children and adolescents within this region. Finally, bullying, by definition, involves different types of repeated aggression with an intent to harm (28). Therefore, the act of bullying itself is subject to debate, because identifying such an act requires an assessment by an outside individual rather than the perceptions of the victim. Moreover, the inclusion of factors such as intention, provocation (29), repetition, and imbalance of power in the universal definition of bullying has been controversial. Because of these issues, an objective measurement of bullying is difficult to obtain (30, 31). Most researchers agree, however, that bullying is a subset of aggressive behaviors defined as negative acts carried out with the intent to cause harm. Therefore, we only used measures of physical aggression in this study due to the difficulty in including proxy assessments (classmates and teachers) about the children/adolescents behavior in the clinical setting. We collected data from parents using a well-known, standardized instrument known as the Child Behavior Checklist (CBCL), and we collected data from adolescents using the Youth Self Report (YSR).

We hypothesized that bullies, victims, and bully-victims all have psychosocial and behavioral problems overseen from their own perspective or that of their parents, and as a result it is necessary to extend treatment to bullies, victims, and bully-victims alike in order to address the roots of the problems and reduce bullying.

## Materials and Methods

### Type of study

Data for this cross-sectional study were collected between January 2009 and January 2010 from seven university-based clinics in a large metropolitan area.

### Participants

We selected participants using the electronic records of patients from seven university-based clinics that primarily serve Mexican-American families with low socioeconomic status (LSES) in a large metropolitan area. We defined LSES as having an income below the poverty level and a household income of <$25,000 per year. We only included in the study patients who were of Mexican-American origin, had LSES, and were between 11 and 16 years old. These criteria excluded <5% of the total available patients. The Institutional Review Board of the Texas Tech University Health Sciences Center approved this study.

During routine pediatric-care visits, parents/caretakers completed several assessments of their child’s behavior, including the CBCL, and the adolescents completed the YSR and a questionnaire including categorization questions as bullies, victims, bully-victims, and uninvolved children. A total of 320 patients satisfied our inclusion criteria. We excluded patients whose mothers’ responses to the CBCL were unavailable and patients who had been previously diagnosed with a chronic mental, neurological, or life-threatening disease or disability. We excluded data that included the responses of fathers or other non-maternal caregivers and data that originated from a patient belonging to Hispanic groups other than Mexican-American. Our final data set included 223 patients (72.9% of the total available patients).

### Measures

We used complete responses to three measures: six self-report questionnaires on adolescent aggression, the YSR, and the CBCL. The six self-report questions, based on the aggression/victimization scale presented in McConville and Cornell (32), assess physical bullying in a clinical setting and categorize individuals into one of four groups: bully (B), victim (V), bully-victim (B + V), or uninvolved (U). The CBCL (completed by the mothers of the adolescents) and the YSR (completed by the adolescents) both assess psychosocial and behavioral problems among adolescents. The CBCL and YSR are nationally standardized instruments used to obtain information about the behavioral and emotional problems of children.

### Aggression/victimization adolescent self-report questions

Three questions assessed the frequency at which an individual participated in aggressive behavior, and three questions assessed the frequency at which an individual suffered from aggressive behavior. The questions that assessed participation in aggressive behavior asked how often the individual hit/kicked, grabbed/shoved, or threatened a weaker person in the preceding 12 months (e.g., “In the last 12 months, how often have you hit or kicked someone weaker than yourself?”). The questions that assessed victimization by aggressive behavior asked how often the individual experienced being hit/kicked, grabbed/shoved, or threatened by someone stronger than themself in the preceding 12 months (e.g., “In the last 12 months, how often have you been hit or kicked by someone stronger than yourself?”). The response options for each item were: never = 0, once = 1, twice = 2, three times = 3, four times = 4, or five or more times = 5. Based on their responses, we categorized the participants into four groups: bully, victim, bully-victim, and uninvolved.

We combined the scores for the three participation questions and the three victimization questions, respectively, to create two subscales. Each subscale had a minimum score of 0 and a maximum score of 15. We placed individuals with scores ≥2 on both subscales, indicating that they had both participated in aggression and been victimized, in the bully-victim group. We placed individuals with scores ≥2 on the participation subscale and scores <2 on the victimization subscale in the bully group. We placed individuals with scores ≥2 on the victimization subscale and <2 on the participation subscale in the victim group. We placed all other individuals in the uninvolved group. We set the cut-off for categorization at values >1 to meet the repetition and imbalance-of-power criteria for bullying (33).

### Child behavior checklist

The CBCL assesses behavioral and emotional problems using 120 questions scored on a three-point scale: 0 = not true, 1 = somewhat or sometimes true, 2 = very or often true. The CBCL is indicative of eight syndromes, of which three (anxious/depressed, withdrawn/depressed, and somatic complaints) load on the internalizing factor and two (rule-breaking and aggressive) load on the externalizing factor. The remaining three syndromes (social problems, thought problems, and attention problems) do not load differentially on either factor. There are three total scores that include internalizing, externalizing, and total problem scales. In addition, the CBCL has six scales that are oriented to the Diagnostic and Statistical Manual of Mental Disorders, fourth Edition (DSM-IV): affective problems, anxiety problems, somatic problems, attention deficit/hyperactivity problems, oppositional defiant problems, and conduct problems (34).

### Youth self report

The YSR provides self-ratings for behavioral problems with questions that are parallel to the questions on the CBCL. It has the same syndromes, factors, and scales as the CBCL and uses the same three-point scale as the CBCL to rate the answer to each question.

We converted the raw scores on the CBCL and the YSR to *T* scores. We defined the “borderline/clinical range” for the internalizing/externalizing factors as a *T* score ≥60 and for the syndrome and DSM scales as a *T* score ≥64. These cut-off scores allow us to dichotomize the borderline/clinical group and the normal group and have been shown to discriminate between children who were referred to mental health services and children who were not referred to mental health services (34).

### Statistical methods

Continuous data were described by the mean and standard deviation (SD), while categorical data were described by the frequency and proportion. The baseline continuous variables were compared using a one-way analysis of variance (ANOVA) and *post hoc* Bonferroni tests, while categorical variables were compared using a Chi-square test. We log-transformed the behavioral scores and compared them among the four groups (B, V, B + V, and U) using a multivariate analysis of variance (MANOVA) adjusted for the confounding variable (gender) followed by a one-way ANOVA and *post hoc* Bonferroni tests. The threshold for statistical significance was *p* < 0.05 in the MANOVA and *p* < 0.006 (Domain I), *p* < 0.025 (Domain II), or *p* < 0.008 (Domain III) in the one-way ANOVA under both the CBCL and YSR scales. We performed all the statistical analyses using SAS 9.3.

## Results

The mean age of the participants was 13 (SD = 1.7) years. Our sample included 109 (48.9%) females and 114 (51.1%) males. Table 1 shows the socio-demographic characteristics of the participants. The distributions of age, language, and insurance status were comparable across the groups. The B + V group had a significantly higher proportion of males than the other groups.

**Table 1:** **Comparison of socio-demographic characteristics of study subjects**.

Variables	Total (*n* = 223)	U (*n* = 99)	B (*n* = 37)	V (*n* = 30)	B + V (*n* = 57)	*p*-Value
Age (years) mean (SD)	13.0 (1.7)	13.1 (1.8)	13.0 (1.8)	12.9 (1.9)	12.9 (1.5)	0.92
Gender
Male *n* (%)	114.0 (51.1)	42.0 (42.4)	16.0 (43.2)	16.0 (53.3)	40.0 (70.2)	0.01
Insurance
Medicaid *n* (%)	139.0 (62.3)	60.0 (60.6)	27.0 (73.0)	17.0 (56.7)	35.0 (61.4)	0.77
Chip *n* (%)	68.0 (30.5)	30.0 (30.3)	8.0 (21.6)	11.0 (36.7)	19.0 (33.3)	
Uninsured *n* (%)	16.0 (7.2)	9.0 (9.1)	2.0 (5.4)	2.0 (6.7)	3.0 (5.3)	
Language
English *n* (%)	136.0 (61.0)	59.0 (59.6)	24 (64.9)	20.0 (66.7)	33.0 (57.9)	0.81

*U, uninvolved; B, bully; V, victim; B + V, bully-victim; SD, standard deviation*.

Tables 2 and 3 show the prevalence of borderline/clinical psychosocial and behavioral problems (excluding DSM scales) in each group based on the results of the CBCL and the YSR, respectively. Across the entire sample, the prevalence of somatic complaints was higher using both scales, followed by aggressive behavior. Internal total problems had a higher prevalence when compared with external total problems. In the borderline and clinical scales, the prevalence of anxious/depressed problems, social problems, attention problems, and aggressive behavior were higher in the V group (based on results from the CBCL), and these problems had highest prevalence in B + V group (based on results from the YSR). The proportion of withdrawn/depressed problems and somatic complaints were highest in the B group according to the CBCL and highest in the B + V group according to the YSR. Thought problems were more prevalent in B group in the CBCL and YSR. Rule-breaking was more prevalent in the B + V group according to the CBCL and more prevalent in the V group according to the YSR. The V group had the highest prevalence of internalizing, externalizing, and total scales according to the CBCL, and these scores were more prevalent in the B + V group according to the YSR.

**Table 2:** **Prevalence of psychosocial and behavioral problems classified by CBCL**.

Variables	Total (*n* = 223)	U (*n* = 99)	B (*n* = 37)	V (*n* = 30)	B + V (*n* = 57)
Anxious/depressed *n* (%)	16.0 (7.2)	4.0 (4.0)	3.0 (8.1)	4.0 (13.3)	5.0 (8.8)
Withdrawn/depressed *n* (%)	15.0 (6.7)	3.0 (3.0)	5.0 (13.5)	3.0 (10.0)	4.0 (7.0)
Somatic complaints *n* (%)	28.0 (12.6)	11.0 (11.1)	7.0 (18.9)	3.0 (10.0)	7.0 (12.3)
Social problems *n* (%)	13.0 (5.8)	2.0 (2.0)	2.0 (5.4)	5.0 (16.7)	4.0 (7.0)
Thought problems *n* (%)	19.0 (8.5)	6.0 (6.1)	6.0 (16.2)	4.0 (13.3)	3.0 (5.3)
Attention problems *n* (%)	19.0 (8.5)	4.0 (4.0)	2.0 (5.4)	5.0 (16.7)	8.0 (14.0)
Rule-breaking *n* (%)	12.0 (5.4)	1.0 (1.0)	2.0 (5.4)	3.0 (10.0)	6.0 (10.5)
Aggressive behavior *n* (%)	20.0 (9.0)	3.0 (3.0)	1.0 (2.7)	7.0 (23.3)	9.0 (15.8)
Internalizing *n* (%)	34.0 (15.3)	10.0 (10.1)	8.0 (21.6)	7.0 (23.3)	9.0 (15.8)
Externalizing *n* (%)	30.0 (13.5)	7.0 (7.1)	3.0 (8.1)	8.0 (26.7)	12.0 (21.1)
Total *n* (%)	36.0 (16.1)	10.0 (10.1)	6.0 (16.2)	8.0 (26.7)	12.0 (21.1)

*U, uninvolved; B, bully; V, victim; B + V, bully-victim*.

**Table 3:** **Prevalence of psychosocial and behavioral problems classified by YSR**.

Variables	Total (*n* = 223)	U (*n* = 99)	B (*n* = 37)	V (*n* = 30)	B + V (*n* = 57)
Anxious/depressed *n* (%)	16.0 (7.2)	2.0 (2.0)	4.0 (10.8)	1.0 (3.3)	9.0 (15.8)
Withdrawn/depressed *n* (%)	15.0 (6.7)	2.0 (2.0)	2.0 (5.4)	2.0 (6.7)	9.0 (15.8)
Somatic complaints *n* (%)	24.0 (10.8)	4.0 (4.0)	6.0 (16.2)	3.0 (10.0)	11.0 (19.3)
Social problems *n* (%)	13.0 (5.8)	2.0 (2.0)	3.0 (8.1)	0.0 (0.0)	8.0 (14.0)
Thought problems *n* (%)	13.0 (5.8)	3.0 (3.0)	5.0 (13.5)	1.0 (3.3)	4.0 (7.0)
Attention problems *n* (%)	9.0 (4.0)	3.0 (3.0)	1.0 (2.7)	0.0 (0.0)	5.0 (8.8)
Rule-breaking *n* (%)	9.0 (4.0)	2.0 (2.0)	0.0 (0.0)	3.0 (10.0)	4.0 (7.0)
Aggressive behavior *n* (%)	18.0 (8.1)	4.0 (4.0)	2.0 (5.4)	2.0 (6.7)	10.0 (17.5)
Internalizing *n* (%)	40.0 (17.9)	4.0 (4.0)	12.0 (32.4)	4.0 (13.3)	20.0 (35.1)
Externalizing *n* (%)	32.0 (14.4)	6.0 (6.1)	3.0 (8.1)	7.0 (23.3)	16.0 (28.1)
Total *n* (%)	33.0 (14.8)	5.0 (5.1)	6.0 (16.2)	5.0 (16.7)	17.0 (29.8)

*U, uninvolved; B, bully; V, victim; B + V, bully-victim*.

Tables 4A,B show the gender-adjusted MANOVA and one-way ANOVA results comparing each domain of the CBCL scores. Each domain of the CBCL and YSR was found to be significantly different among the groups even after adjusting for gender. The one-way ANOVA results show the comparison of the individual raw scores in each domain of the CBCL and YSR separately. In Domain I under the CBCL scale, only rule-breaking and aggressive behavior were found to be different among the four groups. In Domain II, the scores for both internalizing and externalizing problems were significantly different among the groups. The scores for total problems were also found to be different among the groups. Under Domain III, DSM affective, DSM oppositional, and DSM conduct were found to be significantly different among the groups.

**Table 4:** **(A) Adjusted comparisons of psychosocial and behavioral problems according to CBCL raw scores among the groups. (B) *Post hoc* comparisons of scores obtained using CBCL**.

(A)						

Variables	U	B	V	B + V	MANOVA	ANOVA
	
	Mean (SD)	Mean (SD)	Mean (SD)	Mean (SD)	*p*-Value	*p*-Value
Domain I					0.02	
Anxious/depressed	2.0 (2.7)	3.2 (3.1)	3.4 (4.0)	2.4 (2.5)		0.05
Withdrawn/depressed	1.2 (1.7)	2.1 (2.7)	1.9 (2.4)	1.6 (2.1)		0.12
Somatic complaints	1.6 (2.2)	2.6 (2.9)	2.4 (2.9)	1.9 (2.0)		0.03
Social problems	1.2 (1.7)	2.1 (2.8)	2.6 (3.1)	1.8 (2.2)		0.01
Thought problems	1.2 (1.6)	2.7 (4.1)	1.7 (3.0)	1.4 (1.7)		0.07
Attention problems	2.3 (3.1)	2.9 (3.5)	3.9 (4.8)	3.7 (4.0)		0.22
Rule-breaking	1.1 (1.7)	2.2 (3.0)	2.7 (4.3)	1.9 (2.9)		<0.0001
Aggressive behavior	2.8 (3.5)	3.8 (4.7)	7.0 (6.7)	5.6 (5.6)		<0.0001
Domain II					<0.0001	
Internalizing	4.8 (5.4)	8.0 (7.3)	7.8 (8.4)	5.9 (5.2)		0.01
Externalizing	3.9 (4.8)	6.0 (7.3)	9.7 (10.2)	7.5 (8.0)		<0.0001
Domain III					<0.0001	
DSM affective	1.2 (2.1)	2.6 (3.3)	2.4 (3.3)	1.6 (2.0)		<0.0001
DSM anxiety	1.2 (1.6)	2.0 (1.8)	1.7 (1.7)	1.4 (1.7)		0.06
DSM somatic	1.2 (1.7)	1.7 (1.9)	1.8 (2.1)	1.2 (1.6)		0.11
DSM ADH	2.0 (2.5)	2.5 (2.5)	3.2 (3.9)	3.0 (3.4)		0.28
DSM oppositional defiant	1.6 (1.9)	2.1 (2.0)	3.0 (2.6)	3.0 (2.6)		<0.0001
DSM conduct	0.9 (1.7)	2.0 (3.4)	3.3 (4.7)	2.3 (3.8)		<0.0001
Total problems	15.7 (15.5)	24.6 (23.8)	29.3 (29.8)	23.2 (18.3)		<0.0001

**(B)**						

**Variables**	**U vs. B**	**U vs. V**	**U vs. B + V**	**B vs. V**	**B vs. B + V**	**V vs. B + V**

Rule-breaking	0.03	0.03	NS	NS	NS	NS
Aggressive behavior	NS	<0.0001	0.01	NS	NS	NS
Internalizing	0.02	NS	NS	NS	NS	NS
Externalizing	NS	<0.0001	0.03	NS	NS	NS
DSM affective	<0.0001	NS	NS	NS	NS	NS
DSM oppositional defiant	NS	0.02	0.01	NS	NS	NS
DSM conduct	NS	<0.0001	NS	NS	NS	NS
Total problems	0.02	NS	NS	NS	NS	NS

*U, uninvolved; B, bully; V, victim; B + V, bully-victim; SD, standard deviation; NS, not significant*.

The *post hoc* analysis following the ANOVA revealed that the mean scores for rule-breaking (*p* = 0.0274), aggressive behavior (*p* = 0.002), DSM oppositional (*p* = 0.0202), and DSM conduct (*p* = 0.002) were significantly higher in the V group than in the U group. The mean rule-breaking score was also significantly higher in the B group than in the U group. The mean aggressive behavior score was significantly higher in the B + V group than in the U group. The mean DSM affective score was significantly higher in the B group than in the U group, while the mean DSM oppositional score was significantly higher in the V and B + V groups than in the other groups. The internalizing problems score was significantly different between the B group and the U group, while the externalizing problems score was significantly different in the V and B + V groups than in the U group, even after adjusting for gender (Table 4B).

Tables 5A,B show the results of the gender-adjusted MANOVA and one-way ANOVA comparing each domain of the YSR scores. The MANOVA results demonstrate that the score for each domain of the YSR was significantly different among the groups after adjusting for gender. After adjustment for multiple comparisons, the ANOVA results showed that all of the YSR scores were significantly different among the groups. All of the mean scores were significantly higher in the B + V group than in the U group. All of the mean scores except attention, DSM somatic, DSM adherence, and DSM conduct were higher in the B group than in the U group. The rule-breaking, aggressive behavior, DSM oppositional, and DSM conduct scores were significantly higher in the V group than in the U group. The internalizing problems score was significantly different in the B and B + V groups than in the U group, while the B, V, and B + V groups all had higher externalizing problems scores than the U group. The internalizing problems score was also significantly different between V and B + V groups. In addition, the anxious/depressed, withdrawn, social problem, and aggressive behavior scores were significantly higher in the B + V group than in the V group. The social problem score was significantly higher in the B + V group than in the B group, while the DSM conduct score was higher in the B + V group than in the B group (Table 5B).

**Table 5:** **(A) Adjusted comparison of psychosocial and behavioral problems according to YSR raw scores. (B) *Post hoc* comparisons of raw scores obtained using YSR**.

(A)						

Variables	U	B	V	B + V	MANOVA	ANOVA
	
	Mean (SD)	Mean (SD)	Mean (SD)	Mean (SD)	*p*-Value	*p*-Value
Domain I					<0.0001	
Anxious/depressed	2.3 (3.0)	4.8 (3.9)	2.8 (3.0)	5.2 (4.5)		<0.0001
Withdrawn/depressed	1.6 (1.9)	3.0 (2.3)	2.6 (2.4)	4.1 (2.8)		<0.0001
Somatic complaints	2.0 (2.7)	3.9 (3.8)	2.8 (2.9)	4.0 (3.4)		<0.0001
Social problems	1.6 (1.9)	3.5 (3.2)	1.8 (1.9)	3.6 (3.2)		<0.0001
Thought problems	2.0 (2.6)	4.1 (3.2)	2.9 (3.5)	4.1 (3.3)		<0.0001
Attention problems	3.2 (2.9)	4.4 (3.0)	4.0 (2.7)	5.4 (3.3)		<0.0001
Rule-breaking	1.6 (2.2)	2.8 (2.1)	3.7 (3.4)	4.4 (3.5)		<0.0001
Aggressive behavior	3.7 (3.6)	5.4 (3.5)	6.7 (4.7)	8.5 (5.7)		<0.0001
Domain II					<0.0001	
Internalizing	5.9 (6.5)	11.7 (8.0)	8.2 (6.6)	13.3 (9.3)		<0.0001
Externalizing	5.3 (5.3)	8.2 (4.7)	10.5 (7.0)	12.8 (8.1)		<0.0001
Domain III					<0.0001	<0.0001
DSM affective	1.8 (2.4)	3.8 (2.7)	3.1 (3.2)	4.7 (3.9)		
DSM anxiety	1.6 (2.1)	2.9 (2.5)	1.5 (1.8)	2.5 (2.1)		<0.001
DSM somatic	1.4 (1.9)	2.4 (2.6)	1.9 (2.2)	2.5 (2.4)		<0.001
DSM ADH	3.6 (2.8)	3.9 (2.5)	4.0 (2.8)	5.2 (3.0)		<0.001
DSM oppositional defiant	1.9 (1.8)	3.0 (1.8)	2.8 (1.6)	3.6 (2.1)		<0.0001
DSM conduct	1.7 (2.4)	2.6 (2.5)	3.9 (3.2)	4.8 (3.9)		<0.0001
Total problems	21.6 (17.3)	35.9 (18.9)	31.8 (19.6)	44.4 (23.9)		<0.0001

**(B)**						

**Variables**	**U vs. B**	**U vs. V**	**U vs. B + V**	**B vs. V**	**B vs. B + V**	**V vs. B + V**

Anxious/depressed	<0.001	NS	<0.0001	NS	NS	0.05
Withdrawn/depressed	<0.001	NS	<0.0001	NS	NS	0.03
Somatic complaints	<0.001	NS	<0.0001	NS	NS	NS
Social problems	<0.001	NS	<0.0001	0.04	NS	<0.001
Thought problems	<0.001	NS	<0.0001	NS	NS	NS
Attention problems	NS	NS	<0.0001	NS	NS	NS
Rule-breaking	<0.001	<0.001	<0.0001	NS	NS	NS
Aggressive behavior	0.01	<0.001	<0.0001	NS	NS	NS
Internalizing	<0.0001	NS	<0.0001	NS	NS	0.04
Externalizing	<0.001	<0.0001	<0.0001	NS	NS	NS
Total problems	<0.0001	0.01	<0.0001	NS	NS	NS
DSM affective	<0.0001	NS	<0.0001	NS	NS	NS
DSM anxiety	<0.001	NS	0.0103	NS	NS	NS
DSM somatic	NS	NS	0.0003	NS	NS	NS
DSM ADH	NS	NS	0.0032	NS	NS	NS
DSM oppositional defiant	<0.001	<0.001	<0.0001	NS	NS	NS
DSM conduct	NS	<0.001	<0.0001	NS	0.01	NS

*U, uninvolved; B, bully; V, victim; B + V, bully-victim; SD, standard deviation; NS, not significant*.

In summary, the adjusted scores on both scales showed that rule-breaking, aggressive behavior, DSM oppositional, and DSM conduct scores were significantly elevated in the V group, while DSM affective scores was significantly elevated in the B group. According to the YSR, the B + V group had significantly higher scores than the U group for each variable. The social problems score was elevated in the B + V group compared with the U, B, and V groups. In general, all *post hoc* analyses indicated that differences were statistically significant between the U group and at least one of the B, V, or B + V groups. However, no significant differences were observed among the B, V, and B + V groups according to CBCL. Most of the YSR scores were found to be different in B, V, or B + V groups as compared with U group. Few scores were found to be different between the B + V group and either the B or V group.

Table 6 shows the differences in the total problems scores according to different combinations of groups separately for the CBCL and the YSR. The smallest difference between the groups according to both scales was that between the B and V groups. According to the CBCL, the difference between the total problems scores of the B and V groups was 0.01 units (95% CI: −0.62, 0.63). According to the YSR, the difference between the total problems scores of the B and V groups was 0.19 units (95% CI: −0.27, 0.66). If we consider a clinically meaningful difference between the groups to be a difference of at least 0.5 units, then we can conclude that there was no difference between the B + V and B groups or between the B + V and V groups according to the CBCL (because the upper limits of the CIs are <0.5). We can also conclude that there was a significant difference between the B + V and U groups according to the YSR, because the lower limit of the CI is >0.5. In conclusion, there was no difference between the B + V group and either the B group or the V group according to the CBCL, and there was a significant difference between the B + V group and the U group according to the YSR.

**Table 6:** **Comparison of total problems according to different groups**.

Group differences	CBCL			YSR		
	Mean	95% Confidence interval		Mean	95% Confidence interval	
U vs. B	−0.11	−0.64	0.43	0.20	−0.20	0.61
U vs. V	−0.10	−0.67	0.47	0.40	−0.03	0.83
U vs. B + V	0.44	0.02	0.87	0.86	0.54	1.17
B vs. V	0.01	−0.62	0.63	0.19	−0.27	0.66
B vs. B + V	0.55	0.06	1.04	0.65	0.29	1.02
V vs. B + V	0.54	0.01	1.07	0.46	0.06	0.86

## Discussion

We hypothesized that bullies, victims, and bully-victims suffered from emotional and behavioral problems. We assessed each group based on their responses to the YSR and the responses of their parents to the CBCL. These standardized instruments provided results corresponding to different behavioral and emotional problems including syndrome, total, and DSM scales.

Most of the differences that we found among the groups were between the U group and one or more of the B, V, and B + V groups. We found few significant differences among the B, V, and B + V groups. The differences that we found based on the YSR with regard to emotional problems included withdrawn/depressed, social problems, and DSM somatic problems. We found no *Post hoc* significant differences among the B, V, and B + V groups based on the CBCL. Thus, both the mothers (CBCL) and the youths (YSR) were in agreement that children who were uninvolved in bullying generally experience fewer behavioral and emotional problems when compared with children who were involved in bullying. We did not observe much similarity between the responses of mothers (CBCL) and the responses of youths (YSR) within the B, V, and B + V groups. For example, according to the youths, the B + V group had the most emotional and behavioral problems while the B group had more behavioral problems and the V group had more emotional problems. According to the mothers, the B and V groups had the most emotional problems and the B and B + V groups had the most behavioral problems. Discrepancies between the responses of the parents and the responses of the youths have been reported in different studies (35–38). These differences between the perspective of the adolescent and that of the mother are very relevant when we consider the importance of the youth’s impression about his or her own social and emotional behaviors.

In this study, the mothers recognized fewer internalizing problems in the V and B + V groups than the youths recognized. This suggests that many of the youths in those groups are more likely to contend with unrecognized depression. Previous studies concluded that discrepant scores on the CBCL and the YSR (35, 39) deserve special notice when the youth scores are higher than the parent scores, particularly on the internalizing behaviors. The differences observed in our study could be caused by behaviors that are visible to the parent and different from the adolescent’s regular behaviors, giving the mothers a different perspective than that of the youths.

Previous research has helped us to understand more about the nature and scope of bullying, as well as how to identify bullies and victims (40). Although bullying is unacceptable under any circumstances, concentrating solely on policies to reduce bullying behaviors, including disciplinary strategies that only impact the aggressor, might not be effective in deterring the recurrence of bullying behaviors. Bullying behavior among children and adults is surrounded by a multidimensional spectrum of factors that are rooted during the first years of life. Persistent aggression at an early age increases the risk of later juvenile delinquency, adult violence, school failure, and peer problems. The interconnections between the behaviors of the victims and the behaviors of the perpetrators suggest that both of these groups are at risk for alcohol and substance abuse, poor academic achievement, depression, suicide, and antisocial behaviors (41). Early-appearing externalizing behaviors disrupt relationships with parents and peers, thereby initiating processes that can maintain or exacerbate children’s behavioral problems. By stigmatizing these behaviors, the problems are nurtured, and policies to stop these behaviors, at best, only temporarily hide the effects of these behaviors. To stop “bullying behaviors,” it is necessary to end these behaviors by addressing the roots of the problem at an early age, as well as consider the fact that bullies are children with emotional and behavioral problems who can benefit from treatment. Increasing access to treatment may be beneficial for bullies, victims, and bully-victims alike, and it can provide a different approach to solving the problem of bullying.

This research has a number of limitations that can be addressed in future work. This study is cross-sectional; a longitudinal perspective could shed more light on the development of relationships. Our classification scheme was based only on physical bullying rather than on the full spectrum of bullying. We only used adolescent self-report data and did not include direct observations or teacher and peer reports. We did not validate the self-report victimization questions against other measurements; instead we compared the results with those from the CBCL and YSR, which target a wider spectrum of behaviors. The sample size of some of the groups including the victims is reduced, however, it should be pointed out that our intention was not to determine prevalence of groups but instead to look at the similitude of emotional and behavioral problems between the groups. Because of the limited sample size of bullies and victims groups, it will be important to conduct a further investigation with a larger and more diverse sample. Our population sample was composed entirely of Mexican-American individuals with low SES, which may limit the generalizability of the results. However, despite these limitations, we believe this study can be considered an initial step in looking at the problems of bullying from a different perspective, with enough evidence to consider these findings.

In summary, we found that groups of bullies, victims, and bully-victims experienced a panoply of problems that require attention. The problems were similar among the groups according to the assessments of youths and mothers. It was interesting to compare the measure completed by youth (YSR) to that completed by parents (CBCL). Although the adolescent’s psychosocial and behavioral problems agreed with previous reports from adolescents, results from parents do not agree with these previous reports, suggesting that further studies in this area are warranted. Our study suggests that most of the underlying emotional and behavioral problems were not statistically different among the bullies, victims, and bully-victims in our study population. Therefore, treatment should not be focused only on the victims; it is equally important for the other groups as well.

This study is grounded in the evidence that suggests overlaps between different forms of violence and victimization. As suggested by Sherry Hamby and John Grych (17) a large number of studies have been generated on child maltreatment, bullying, intimate partner violence, elder abuse, etc., with few systematic efforts to understand connections among them. Despite few exceptions, each field has developed its own conceptual models, knowledge, and strategies for intervention. This focused approach has left a gap in the knowledge between the different forms of interpersonal violence connections across contexts and over the life span in the lives of victims, perpetrators, and those involved in violence as both victims and perpetrators. Our study indicated emotional and behavioral problems among the bullies, victims and bully-victims that need to be addressed and solved by considering bullying behaviors as overlapping effects of exposure to victimization.

Failure to adequately treat the underlying problems experienced by all three groups of individuals could help to seed future generations of bullies, victims, and bully-victims. Including a psychosocial and behavioral perspective should be considered as a valuable strategy to increase the long-term success rates of efforts to eliminate the problem of bullying.

## Conflict of Interest Statement

The authors declare that the research was conducted in the absence of any commercial or financial relationships that could be construed as a potential conflict of interest.
